# Prevalence of haemosporidia in Asian Glossy Starling with discovery of misbinding of *Haemoproteus*-specific primer to *Plasmodium* genera in Sarawak, Malaysian Borneo

**DOI:** 10.1186/s12917-023-03619-y

**Published:** 2023-04-20

**Authors:** Vaenessa Noni, Cheng Siang Tan

**Affiliations:** Center for Tropical and Emerging Diseases, Faculty of Medicine and Health Sciences, Universiti Malaysia Sarawak, 94300 Kota Samarahan, Sarawak Malaysia

**Keywords:** Misbinding, Avian, Plasmodium, Genera-specific primer, Sarawak

## Abstract

**Background:**

*Plasmodium*, *Haemoproteus* and *Leucocytozoon* are three mainly studied blood parasites known to cause malarial and pseudomalarial infections in avian worldwide. Although Sarawak is a biodiversity hotspot, molecular data on blood parasite diversity in birds are absent. The objective of the study is to determine the prevalence of blood parasite in Asian Glossy Starlings (AGS), an urban bird with high population density in Sarawak and to elucidate the phylogenetic relationship with other blood parasite.

**Methods:**

Twenty-nine carcasses of juvenile AGS that were succumbed to death due to window collision were collected around the vicinity of Universiti Malaysia Sarawak. Nested-multiplex and nested PCR targeting the Cytochrome B gene were used to detect *Plasmodium* and *Haemoproteus*, and *Leucocytozoon* respectively. Two primer sets were used for *Haemoproteus* detection to increase detection sensitivity, with one being a genus-specific primer.

**Results:**

Fourteen samples (prevalence rate: 48.28%) were found positive for avian *Plasmodium*. Phylogenetic analysis divided our sequences into five lineages, pFANTAIL01, pCOLL4, pACCBAD01, pALPSIS01 and pALPSIS02, with two lineages being novel. No *Haemoproteus* and *Leucocytozoon* was found in this study. However, *Haemoproteus*-specific primer used amplified our *Plasmodium* samples, making the primer non-specific to *Haemoproteus* only.

**Conclusion:**

This is the first blood parasite detection study on AGS using carcasses and blood clot as sample source in Sarawak. Due to the scarcity of longer sequences from regions with high genetic plasticity, usage of genus-specific primers should be validated with sequencing to ensure correct prevalence interpretation.

**Supplementary Information:**

The online version contains supplementary material available at 10.1186/s12917-023-03619-y.

## Introduction

Avian haemosporidia, consisting of *Plasmodium*, *Haemoproteus* and *Leucocytozoon* genera, are blood obligated protozoans which causes malaria and malaria-like diseases in susceptible avian hosts [1–3]. Disease presentations can range from asymptomatic to potentially fatal depending on haemosporidian lineage and bird species. Its importance came to light after the extirpation of numerous endemic Hawaiian wild birds in the 1900s due to the spread of *Plasmodium relictum* lineage, pGRW4, through the introduction of mosquito vector, *Culex quinquefasciatus* and infected exotic birds [4–6]. To this day, it is considered as the key factor limiting the presence of endemic birds on the island [7]. Since then, routine molecular malarial screening in captive and migratory birds has led to the discovery of vast diversity of avian haemosporidia lineage including *Plasmodium*, *Haemoproteus* and *Leucocytozoon* genera [8–10].

There are two routine methods used for haemosporidian parasites detection, which are microscopic observation of erythrocytic stages and PCR methods to increase the detection sensitivity especially in low parasite density and mixed species or lineage infection [3, 11]. Inclusion of both methods detected the presence of morphospecies and high lineage diversity using the conserved mitochondrial gene such as cytochrome B (*CytB*) and cytochrome oxidase subunit 1 (*COI*) [12]. PCR protocols for detecting avian haemosporidian comprises of consensus PCR protocol, which is able to simultaneously amplify the conserve gene of the three avian haemosporidian genera [13–18]. However, the limitation of the available nested PCR protocols for avian malaria detection is its inability to immediately discriminate mixed genus or lineages without requiring further downstream methods such as recombinant DNA technology or next generation sequencing (NGS) [16, 19, 20]. To rectify this problem, multiplexing protocols has been deduced to increase specificity in detection of mixed genera infection without requiring downstream protocols for genera identification and reduce biased amplification of genera with higher template copy especially in the context of wildlife samples [21, 22].

In Malaysia, the first avian haemosporidian prevalence study was done in three states, Sarawak, Johor and Pahang [23]. Utilising microscopy as detection method, a concise checklist of haemosporidian parasites from wild birds based of morphology was produced. However, this limited the data up to species level for detected *Plasmodium* and *Haemoproteus* species, whereas up to genus only for *Leucocytozoon*. As microscopic method was used, molecular information such as lineages from this study was not available and is insufficient for comparison with current available data [24]. Similar to previous study mentioned, another malarial prevalence study was also conducted but focused in Selangor, Malaysia which included both microscopy and PCR methods for detection [11]. Of the 30.3% positive samples, 16 parasite lineages were identified with 13 novel lineages (*Haemoproteus*: 10 lineages; *Plasmodium*: 3 lineages). Among the three previously described lineage, two (hCOLL2 and hYWT2) were previously detected in Gansu province, China [25]. This suggests that the lineage has a broad distribution or that transmission of malarial parasite from migratory birds to local birds may be possible [26–28].

In Sarawak, Asian Glossy Starling (AGS; *Aplonis panayensis*; Family: Sturnidae) is an urban invasive bird with highest abundance [29, 30]. Its successful invasion in urban setting is attributed to its opportunistic nature to compete for nesting sites and ability to scavenge food resource from its surrounding apart from fruits such as *Ficus benjamina* [29–31]. Majority of studies relating to AGS in Malaysia revolves around roosting site [31, 32], diet composition [29] and characterization of zoonotic endoparasite for public health surveillance [33]. To date, no malarial detection has been done in AGS in Malaysia, with only one prevalence data from Singapore through microscopy characterization obtaining zero *Haemoproteus* prevalence [34]. With this, we took the opportunity to conduct a malarial prevalence study on deceased juvenile AGS that were succumbed to urban mortality due to window collision.

The aim of this study is to investigate the avian malarial diversity using molecular methods in AGS in Sarawak, Malaysian Borneo and the phylogenetic relationship with other haemosporidia around the world.

## Materials and methods

### Sample collection

Twenty-nine blood clot samples were obtained from deceased juvenile AGS that were found in the vicinity of Universiti Malaysia Sarawak (UNIMAS), Sarawak from October to December 2021. The presumed cause of death of the birds were due to window smashing during evening foraging. No further necropsy observation was done. Identification of juvenile birds were identified through plumage colouration as described by Myers [35]. Blood clots were taken from the heart of individual bird and kept in 200uL of lysis buffer from the High Pure Viral Nucleic Acid Extraction kit (Roche, Switzerland) and phosphate buffered saline (PBS) respectively in 1:1 ratio at -20 °C until processed.

### Haemosporidian amplification

**Fig. 1:**
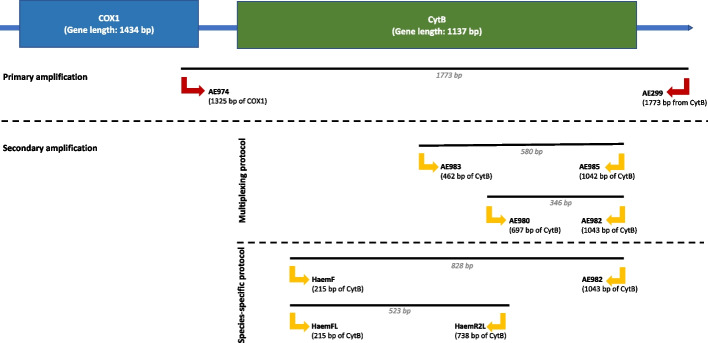
Diagram of mitochondrial genes (*COX1* and *CytB* gene) and binding sites of primer sets used in this study. AE974/AE299 primer sets was used for consensus amplification of the three genera. AE980/AE982 and AE983/AE985 primer sets were used for multiplexing PCR for *Haemoproteus* and *Plasmodium* detection respectively. HaemF/AE982 and HaemFL/HaemR2L primer set was used for genus-specific detection of *Haemoproteus* and *Leucocytozoon* respectively. Expected amplicon size of each primer sets are indicated below the primer length and italicised. Position of nucleotide on mitochondrial gene involved in this study are mentioned below each gene name

Genomic DNA (gDNA) extraction was done using High Pure Viral Nucleic Acid Extraction kit (Roche, Switzerland). Molecular identification was done using the nested and nested-multiplex PCR protocol targeting the *CytB* gene of *Plasmodium*, *Haemoproteus* and *Leucoytozoon* (see Fig. 1 for primer binding site). All primers used were previously designed by Pacheco et al. [22], except for the nested amplification primer set for *Leucocytozoon* which was designed by Hellgren and colleagues [16]. (see Additional file; Fig. 1 for haemosporidian detection workflow).

Primary amplification PCR conditions were done using primer set AE974/AE299 with PCR conditions are as followed: 2 min at 94 °C; 36 cycles of 94 °C for 1 min; 56 °C for 1 min, and 72 °C for 1 min; with final extension for 10 min at 72 °C. Secondary amplification for *Haemoproteus* and *Plasmodium* detection was done using the AE980/AE982 and AE983/AE985 primers with expected band size of 346 and 580 bp respectively. Further validation for negative *Haemoproteus* genus detection through multiplexing was done using specific primer HaemF/AE982, with expected band size of 820 bp to obtain additional positives. PCR conditions for the two secondary nested PCR are identical to the primary amplification but performed with varied with different annealing temperature (refer to Additional file: Table S1). On the other hand, *Leucocytozoon* detection was done using primer HaemFL/HaemR2L with expected size of 523 bp with PCR conditions of: 3 min at 94 °C, 35 cycles of 30 s at 94 °C, 30 s at 50 °C and 45 s at 72 °C, and 72 °C for 10 min. Master mix used for all amplifications contains as followed: 2uL of gDNA/PCR product, 20 pmol of primer, 3 mM MgCl_2_, dNTPs, 1X DreamTaq buffer, DreamTaq polymerase, and added with ultra-pure water until 50uL per reaction was achieved. Amplicons for both primary and secondary amplicons were resolved on 1.5% gel in 1X TBE buffer with 10ug/mL ethidium bromide (Promega, USA) excised and sequenced using primer HaemF and AE982 through Sanger sequencing (1^st^ Base, Malaysia). Sequences that were found to have multiple peaks in their chromatographs were subjected thymine-adenine cloning (TA cloning), by ligation into pJet2.1 vector and transformed into chemically competent *Escherichia coli* DH5α (Thermo Scientific, USA). Five bacterial colonies were randomly picked and sequenced using vector primers.

Primary and secondary amplification primers were chosen based on its previously proven high sensitivity rate. Primary amplification using primer set AE974/AE299 was found to have a sensitivity rate of 81.81% for all three avian haemosporidian genera (parasitemia intensity as low as 0.62% for *Plasmodium*; 0.05% for *Haemoproteus*; 0.01% for *Leucocytozoon*). Whereas, secondary amplification using primer set AE980/AE982 and AE983/AE985 for multiplexing protocol had a sensitivity rate of 100% and 94.4% respectively with the ability to amplify parasitemia as low as 0.01% for both haemosporidian genera [22]. On the other hand, primer set HaemFL/HaemRL2 was able to amplify *Leucocytozoon* with parasitemia as low as 0.001% (equivalent to 1 parasite to 10,000,000 erythrocytes) through nested PCR [16].

### Phylogenetic analysis

Raw sequences were analysed using the Basic Local Alignment Search Tool software (BLAST) (https://blast.ncbi.nlm.nih.gov/Blast.cgi) and MalAvi (http://130.235.244.92/MalAvi/) [24] to determine parasite genus and most similar published lineage respectively. Forward and reverse sequences together with its similar matches were then aligned for sequence homology identification. In the case of differences of one or more single nucleotide polymorphism compared to highest matched lineages, sequence was classified as a novel lineage [8, 16]. A multiple alignment of homologous sequences and related available sequences from GenBank and MalAvi was executed using Clustal Omega (https://www.ebi.ac.uk/Tools/msa/clustalo/) [36]. Bayesian v3.2.7a using the GTR + G + I model as suggested by jModelTest software v.2.1.6. Both jModelTest and MrBayes software were run in CIPRES Science Gateway [37]. Pairwise distance to determine the divergence between positive samples with matched lineages were calculated using the Tamura-Nei model of substitution, with all substitution weighted equally, implemented in MEGA11 [38].

## Result

### Molecular detection of avian parasites in clotted blood

**Fig. 2:**
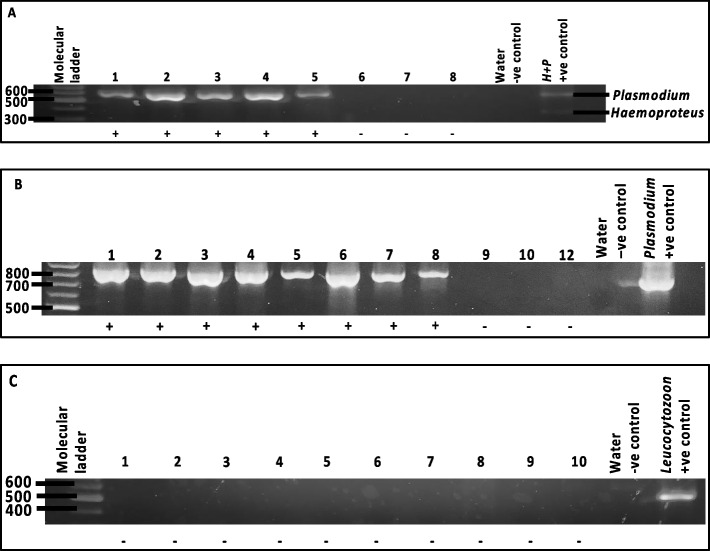
Cropped electrophoresis gel image of **A**. nested-multiplex PCR amplification of the CytB gene of avian *Plasmodium* and *Haemoproteus* using primer set AE980/AE982 and AE983/AAE985 respectively, producing amplicon of 580 bp only; **B**. nested PCR amplification of the CytB gene of avian *Plasmodium* using *Haemoproteus*-specific primer set HaemF/AE982 producing amplicon of 820 bp; **C**. nested PCR amplification of CytB gene of avian *Leucocytozoon* using primer set HaemFL/HaemR2L producing amplicon of 523 bp. (see Additional files 3, 4, and 5: Figure S2 – S4). Visualisation of PCR amplifications for nested-multiplex and nested protocol were done on different gels. The last two lanes on the gel contains the negative (water) and positive control for each primer set. Expected band size of amplicons were based on the 100 bp molecular weight ladder, indicated by the bold line. Positive and negative interpretations are indicated below each band

**Fig. 3:**
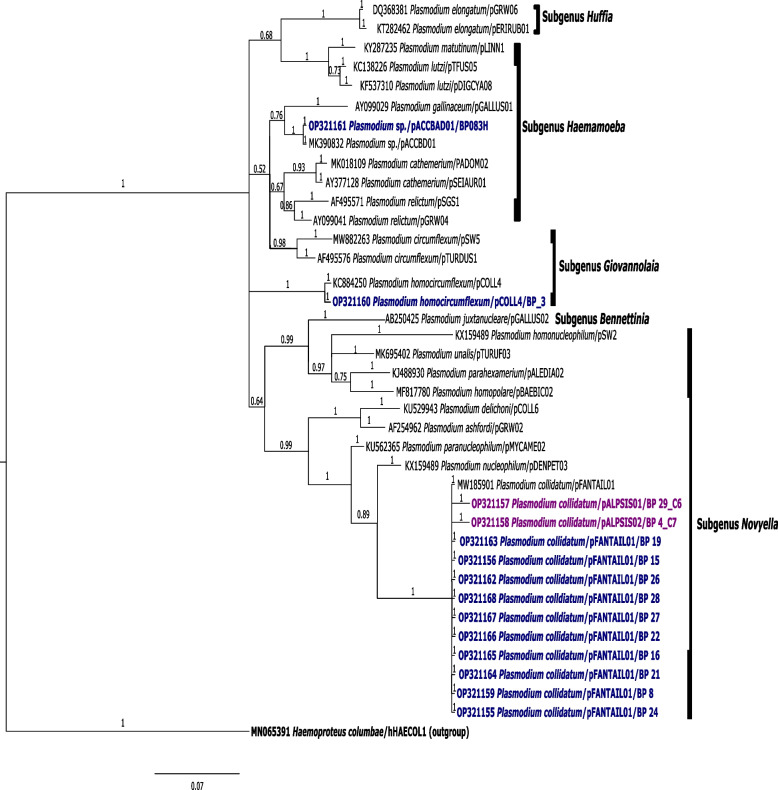
Bayesian inference phylogenetic tree of positive samples and lineages with the highest similarity from MalAvi database and GenBank using 479 bp sequences of the CytB gene. Nodal support values near branches indicate posterior clade probabilities. Samples matched to *P. homocircumflexum* (GenBank accession: KY653784; pCOLL4), *Plasmodium* sp. (MF442584; pACCBAD01) and *P. collidatum* (DQ212193; pFANTAIL01, pALPSIS01, pALPSIS02). *H. columbae* (MN065391) was used as the outgroup in the phylogenetic tree. Bolded in dark blue are lineages that were detected in previous studies; bolded in purple are novel lineages detected in this study

Of the 29 samples, 14 samples (48.28%) were found positive for avian *Plasmodium* through multiplexing, producing a 580 bp amplicon size (Fig. 2). To reconfirm the zero amplification of *Haemoproteus* through multiplexing, *Haemoproteus*-specific primer set was used. *Haemoproteus*-specific primers amplified 820 bp band to the 14 samples previously positive for *Plasmodium* (Fig. 3), suggesting mixed infection. However, nucleotide sequencing of excised amplicons revealed the identified as *Plasmodium*, revising the prevalence of 48.28% haemosporidia to be solely made up of *Plasmodium* lineages only. Of the fourteen amplified samples, five were detected positive by primary PCR indicting high parasitemia in these individuals.

### The analysis of the sequences from multiplex PCR

The analysis of the primer binding sites of the *Plasmodium* sequences revealed that 12 of 14 sequences had a ‘G’ to ‘T’ nucleotide substation at the 3’-end of the reverse primer, AE982 (position 1043 bp) (Table 1). Apart from that, the remaining two sequences had a ‘T’ nucleotide mismatch to the reverse primer AE982, inferring that G/T misbinding occurred during PCR. Further troubleshooting through gradient PCR with annealing temperature ranging from 45 °C to 55 °C did not improve binding specificity of primer to template.

**Table 1:** Binding site of the last 10 nucleotides of reverse primer, AE982 to positive samples and sequences with highest similarity percentage. First row of the table represents the position of the nucleotide on the CytB gene. Two samples were observed with G/T misbinding on the 3’-end of reverse primers post sequencing whereas twelve samples were observed to have exact sequence with *Haemoproteus*-specific primer. Possible insertion or deletion at binding site were reconfirmed by sequencing visible amplicons of 1773 bp size under UV light from the first round consensus PCR. Highlighted yellow: AE982 reverse primer sequence from 5’-end; Highlighted blue: sequence at primer binding site of positive samples; Highlighted green: sequence at primer binding site of reference with highest match; Highlighted orange: mismatch nucleotides to primer sequence. Dots ‘⋅’ represents similar nucleotide with primer nucleotide

Position at *CytB* gene	5'	1034	1035	1036	1037	1038	1039	1040	1041	1042	1043	3'
Reverse primer AE982 (*Haemoproteus*)		**A**	**T**	**R**	**W**	**A**	**R**	**A**	**T**	**A**	**G**	
OP321162 pFANTAIL01_BP_26		・	・	**G**	**T**	・	**G**	・	・	・	**T**	
OP321157 pALPSIS01_BP_29_C6		・	・	**A**	**T**	・	**G**	・	・	・	**T**	
OP321166 pFANTAIL01_BP_22		・	・	**G**	**T**	・	**G**	・	・	・	・	
OP321165 pFANTAIL01_BP_16		・	・	**G**	**T**	・	**G**	・	・	・	・	
OP321168 pFANTAIL01_BP_28		・	・	**G**	**T**	・	**G**	・	・	・	・	
OP321167 pFANTAIL01_BP_27		・	・	**G**	**T**	・	**G**	・	・	・	・	
OP321159 pFANTAIL01_BP_8		・	・	**G**	**T**	・	**G**	・	・	・	・	
OP321163 pFANTAIL01_BP_19		・	・	**G**	**T**	・	**G**	・	・	・	・	
OP321158 pALPSIS02_BP_4_C7		・	・	**G**	**A**	・	**G**	・	・	・	・	
OP321155 pFANTAIL01_BP_24		・	・	**G**	**T**	・	**G**	・	・	・	・	
OP321156 pFANTAIL01_BP_15		・	・	**G**	**T**	・	**G**	・	・	・	・	
OP321164 pFANTAIL01_BP_21		・	・	**G**	**T**	・	**G**	・	・	・	・	
OP321160 pCOLL4_BP_3		・	・	**G**	**T**	・	**G**	・	・	・	・	
OP321161 pACCBAD01-BP_83		・	・	**G**	**T**	・	**G**	・	・	・	・	
MF442548 *Plasmodium*_MDG_P12		・	・	**G**	**T**	・	**G**	・	・	・	**T**	
GQ395660 *Plasmodium*_LA20TRMB		・	・	**G**	**T**	・	**A**	・	・	・	**T**	
KY653751 *Plasmodium*_OHA1		・	・	**G**	**T**	・	**A**	・	・	・	**T**	
KJ499987 *H. macrovacuolatus*		・	・	**G**	**T**	・	**A**	・	・	・	・	

### Phylogenetic analysis

The haemosporidians found in our study were classified into three clades with medium to high nodal support ranging (> 0.53) corresponding to the studied subgenera in this study. Five lineages were detected from 14 positive samples, with two lineages being novel. Lineages detected highly matched to pCOLL4 (*P. homocircumflexum*; Subgenus: *Giovannolaia;* *n* = 1), pFANTAIL01 (*P. collidatum*; Subgenus: *Novyella*; *n* = 12), pACCBAD01 (*Plasmodium sp.*; Subgenus *Haemamoeba*; *n* = 1) and novel lineages pALPSIS01 and pALPSIS02 (*Plasmodium sp.*; Subgenus: *Novyella*; *n* = 1 respectively) (Fig. 3).

The novel lineages pALPSIS01 and pALPSIS02 were found to be monophyletic to pFANTAIL01, with high nodal support of 1.0 and pairwise distance of 1.09% with difference of one nucleotide at different position proposing the discovered lineages belonging to *P. collidatum*. On the other hand, the undescribed pACCBAD01 lineage found in this study was found to be monophyletic to *P. gallinaceum* (lineage pGALLUS01), belonging to subgenus *Haemamoeba* with medium nodal support of 0.75 and genetic distance of 3.37% proposing lineage pACCBAD01 a possible morphospecies of *P. gallinaceum*.

## Discussion

This study investigated the prevalence of haemosporidians in AGS that were succumbed to death by window smashing in Sarawak, Malaysian Borneo using multiplexing protocol that was previously reconfirmed by Pacheco et al. [22]. Of the 29 carcasses samples, we initially found 14 samples positive for only *Plasmodium* genus through multiplexing PCR and zero *Leucocytozoon*. However, there was discrepancy in the result after PCR reconfirmation was done using *Haemoproteus*-specific primer set which indicated co-infection in positive samples that were previously detected *Plasmodium*-positive. Sanger sequencing of amplicons then reconfirmed that haemopsoridian positive samples harboured *Plasmodium* only.

The investigation demonstrated that *Haemoproteus*-specific primer set produced by Pacheco et al. may not be suitable for rapid genus identification although proven to be highly specific through PCR validation from labs in USA, Lithuania, and Columbia. *Haemoproteus*-specific primer set HaemF/AE982 is made up of primer HaemF, which was previously designed and used in detection of both *Haemoproteus* and *Plasmodium* by Bensch et al. [39], whereas, AE982 primer set was designed to amplify *Haemoproteus*-only species and used in both multiplexing and genus-specific PCR detection. With this information, we can deduce that AE982 reverse primer determines the genus-specificity of the primer. However, we found that chosen binding site for *Haemoproteus* primer compliments *Plasmodium* as well. This may have been overlooked during primer design as lineages from biodiversity hotspot regions with high haemosporidian diversity and genetic plasticity still remains unexplored [22, 40, 41], taking Borneo as an example. Besides, frequently used primer set HaemF/HaemR2 only produces amplicon size of around 479 bp. This limits the submission of longer sequences in public depository domains such as GenBank and MalAvi, making longer reference sequences to improve primer design of genus-specific primers scarce. Apart from that, mismatch of ‘G’ to two of our positive samples containing ‘T’ nucleotide at position 1043 bp of the 3’-end of the primer may indicate that one SNP may not be sufficient to increase specificity of primer set.

On the other hand, multiplexing PCR protocol using primer set AE980/AE982 for *Haemoproteus* and AE983/AE985 for *Plasmodium* detection produced expected amplicon size of 580 bp, detecting *Plasmodium* only in our samples. In the primer set used for multiplexing, we observed that forward primers AE980 and AE983 were responsible for producing the differentiable band size of 346 bp and 580 bp as reverse primers AE982 and AE985 have similar binding site at position 1042 bp and 1043 bp of the CytB gene respectively. Thus, multiple bands can still be observed from samples with mixed *Haemoproteus* and *Plasmodium* infection despite the possibility of reverse primer AE982 cross-annealing to *Plasmodium* species when using the multiplexing protocol as found in our study.

Our findings are in agreement to other haemosporidian detection studies in species of the Sturnidae family in Australia, India and Myanmar, detecting high prevalence of *Plasmodium* [42–44], but these studies also detected the presence of *Haemoproteus* and *Leucocytozoon*. The lack of their presence in our study can be attributed to the availability of biting vectors in urban setting, which also contributes to the dominance of one haemosporidian genera over the other. Mosquitoes (Culicidae), biting midges (*Culicoides*) and black flies (Simuliidae) are biting vectors for avian *Plasmodium*, *Haemoproteus* and *Leucocytozoon* respectively. *Haemoproteus* and *Leucocytozoon* in natural habitats tends to be higher compared to in urban areas [45, 46] as their vectors require stable environment such as soil–water interface and constant running water respectively for breeding grounds [47]. Whereas, Culicidae mosquitoes are able to thrive in urbanised environments, requiring small temporary water bodies which allows proliferation of their numbers and increasing numbers of *Plasmodium* vectors [48]. Secondly, *Plasmodium* dominance can be caused by the strong specificity of parasite to their vertebrate host [41] unlike *Haemoproteus* and *Leucocytozoon* which has been observed to be host-specific unlike its host-generalist *Plasmodium* [13, 43, 49]. Therefore, the absence of non-*Plasmodium* genera in our study may be attributed by the lack of AGS-specific haemosporidian and biting vectors in Sarawak.

From the 14 Plasmodium positive samples, five lineages were detected in this study with two recorded as novel lineages. Firstly, pACCABD01 is an undescribed host-generalist *Plasmodium* lineage first detected in *Accipter badius* and *Halliastur indus* (GenBank accession no.: JN639001; MZ502250) from Thailand [50]. Based on our phylogenetic tree (Fig. 3), we found that pACCBAD01 belongs to subgenus *Haemamoeba*, a morphospecies to *P. gallinaceum* lineage, GALLUS01 which is typically associated with reduced quality and quantity production of poultry meat and eggs and fatal to its host with mortality rate between 80%—90% in its host [51, 52].

On the other hand, the remaining two lineages are, *P. collidatum* pFANTAIL01 and *P. homocircumflexum* pCOLL4 which has been described as host-generalist pathogenic parasite lineage. Susceptible hosts including birds from the Sturnidae family have been shown to develop high parasitemia when infected with the mentioned lineages [24, 44, 53–55]. Experimentally infection of pFANTAIL01 presented development of exo-erythrocytic stages in the kidney, liver, spleen, and lungs of host [55]. Whereas additional histology of the brain in experimental host infected with pCOLL4 exhibited phanerozoites blockage in the brain capillaries suggesting cerebral ischaemia as the main cause of mortality [54]. However, it is unknown as to the possibility of phanerozoites developing in the brain from the *P. collidatum* lineage pFANTAIL01 lineage as it has not been reported.

Juvenile AGS succumbed to death by window collision has been observed as a common occurrence due to it nescience of flying behaviours in urban areas during foraging periods [56, 57]. However, a recent study in Switzerland found developing phanerozoites in the ocular structure of infected fieldfare, *Turdis pilaris* with *P. matutinum* (pLINN1) which was succumbed to death due to collision with a slow-moving vehicle [58]. This suggests the possibility of visual impairments as well in our AGS apart from developing learned behaviour. However, this is still unknown as previous exo-erythrocytic haemosporidian life cycle studies in both naturally and experimentally haemosporidian rarely includes histology of the eye. Future studies should include other organs for histological observation apart from the internal organs and pectoral muscle to further understand the development of exo-erythrocytic stages of avian *Plasmodium* in infected host.

With the high prevalence of parasite found this study, carcasses may be good sample source to understand the diversity of pathogenic haemosporidia in naturally infected host. An Austrian study employed a citizen-scientist approach by collecting carcasses for malarial detection, and demonstrated carcasses as good sample source for the study [59]. However, decomposition state of the carcasses affected the prevalence reported. Thus, this should be taken into consideration to avoid potential degradation of genetic material, imposing false negative through PCR. Next, apart from muscle tissue and blood drops from carcasses [58, 59], blood clot can also be used for extraction of avian haemospordia gDNA. Our protocol did not require the addition of glass beads for further disruption, yielding similar results to de Abreu and colleagues [60] except that the protocol was used to detect presence of *Plasmodium* species infecting non-human primate from blood clot samples.

As sample size of the study was highly dependent on the juvenile AGS that were succumbed to death during fruiting season (October – December), a small number of birds were obtained to conduct this study. Due to the limitation, inclusion of trapping methods such as mist net during non-fruiting months should be considered in future studies to understand the diversity and changes in haemosporidian epidemiology in AGS in Sarawak. With the knowledge of misbinding of *Haemoproteus*-specific primers to *Plasmodium* genus obtained in this study, designing new specific primers should be taken into consideration with priority of including sequences from regions with high biodiversity during primer design stage. However, other multiplexing primers such as those designed by Ciloglu and colleagues [21, 61] can also be considered as an alternative. Although sequences as long as 820 bp were obtained in this study, the specificity of primer to our haemosporidians for the alternative multiplexing primers could not be evaluated due to the non-overlapping binding site of the Ciloglu primers and primers used in this study.

## Conclusion

This is the first blood parasite detection study on Asian Glossy Starling using carcasses as sample source in Sarawak. The complete reliance on genus-specific protocol to identify and differentiate haemosporidia in avian species is not possible at this moment due to the rich nucleotide diversity in avian malaria. A situation made worse with the paucity of sequence data in many regions in the world. However, we demonstrated that HaemF/AE982 can still be used to obtain longer sequences for phylogenetic analysis study. It is recommended that even when using primer set with proven high sensitivity and specificity rate, protocol validation via sequencing should be included to avoid misinterpretation of the prevalence.

## Supplementary Information

**Additional file 1:** **Figure S1.** Flowchart containing the workflow used  for the detection of avian haemosporidians in our study.

**Additional file 2:** **Table S1.** Primer name, sequence and annealing temperature used for the amplification of the three main avian haemosporidians included in our study, Plasmodium, Haemoproteus and Leucocytozoon. Primer length is included in the expected amplicon size.

**Additional file 3:** **Figure S2. **Uncropped electrophoresis gel of nested-multiplex PCR amplification of CytB gene of avian Plasmodium and Haemoproteus using primer set AE980/AE982 and AE983/AE985 producing amplicons of 580bp only. A positive amplification of Haemoproteus is indicated by the 346 bp amplicon in the positive control. Cropped region presented in the manuscript is denoted by the red box and labelled as Fig. 2A.

**Additional file 4:** **Figure S3. **Uncropped electrophoresis gel of nested PCR of amplification of CytB gene of avian Plasmodium using Haemoproteus-specific primer set HaemF/AE982 producing amplicons of 820bp only. Cropped region presented in the manuscript is denoted by the red box and labelled as Fig. 2B.

**Additional file 5:** **Figure S4. **Uncropped electrophoresis gel of nested PCR of amplification of CytB gene of avian Leucocytozoon using nested primer set HaemFL/HaemRL2 producing amplicon of 523 bp. Cropped regions presented in the manuscript is denoted by the red box and labelled as Fig. 2C.

## Data Availability

The dataset generated and analysed during the current study has been made available in GenBank (https://www.ncbi.nlm.nih.gov/) under the Accession Number OP321155 – OP321168.

## Abbreviations

AGS: Asian Glossy Starlings
*CytB*: Cytochrome B
sp.: Species
*COI*: Cytochrome Oxidase 1
sp.: Species
SNP: Single nucleotide polymorphism
*P.*: *Plasmodium*
*H.*: *Haemoproteus*
*L.*: *Leucocytozoon*

## References

[1] Palinauskas V, Valkiunas G, Bolshakov CV, Bensch S (2008). Plasmodium relictum (lineage P-SGS1): effects on experimentally infected passerine birds. Exp Parasitol..

[2] Palinauskas V, Valkiunas G, Križanauskiene A, Bensch S, Bolshakov CV (2009). Plasmodium relictum (lineage P-SGS1): Further observation of effects on experimentally infected passeriform birds, with remarks on treatment with Malarone™. Exp Parasitol.

[3] Valkiunas G. Avian Malaria Parasites and other Haemosporidia. Avian Malar Parasites other Haemosporidia. 2004 Oct 28 [cited 2022 Apr 24]; Available from: https://www.taylorfrancis.com/books/mono/10.1201/9780203643792/avian-malaria-parasites-haemosporidia-gediminas-valkiūnas.

[4] Van Riper III C, Van Riper SG., Goff M. L, Laird M. The Epizootiology and Ecological Significance of Malaria in Hawaiian Land Birds Published by : Ecological Society of America Stable URL : http://www.jstor.org/stable/1942550. the epizootiology and ecological significance of malaria in Hawaiian land birds. Ecol Monogr. 1986;56(4):327–44.

[5] Warner RE (1968). The role of introduced diseases in the extinction of the endemic Hawaiian Avifauna. Condor.

[6] Daszak P, Cunningham AA, Hyatt AD (2000). Emerging infectious diseases of wildlife - threats to biodiversity and human health. Science  (80- ).

[7] Liao W, Atkinson CT, Lapointe DA, Samuel MD. Mitigating future avian malaria threats to Hawaiian forest birds from climate change. 2017;1–25. 10.1371/journal.pone.0168880 PMC5218566 28060848

[8] DeBrock S, Cohen E, Balasubramanian S, Marra PP, Hamer SA (2021). Characterization of the plasmodium and haemoproteus parasite community in temperate-tropical birds during spring migration. Int J Parasitol Parasites Wildl.

[9] Pigeault R, Vézilier J, Cornet S, Zélé F, Nicot A, Perret P (2015). Avian malaria: a new lease of life for an old experimental model to study the evolutionary ecology of Plasmodium. Philos Trans R Soc B Biol Sci.

[10] Valkiunas G, Ilgunas M, Bukauskaite D, Fragner K, Weissenböck H, Atkinson CT (2018). Characterization of plasmodium relictum, a cosmopolitan agent of avian malaria. Malar J.

[11] Ivanova K, Zehtindjiev P, Mariaux J, Georgiev BB (2015). Genetic diversity of avian haemosporidians in Malaysia: cytochrome b lineages of the genera plasmodium and haemoproteus (Haemosporida) from Selangor. Infect Genet Evol.

[12] Valkiunas G, Iezhova TA (2018). Keys to the avian malaria parasites. Malar J.

[13] Beadell JS, Gering E, Austin J, Dumbacher JP, Peirce MA, Pratt TK (2004). Prevalence and differential host-specificity of two avian blood parasite genera in the Australo-Papuan region. Mol Ecol.

[14] Bell JA, Weckstein JD, Fecchio A, Tkach VV (2015). A new real-time PCR protocol for detection of avian haemosporidians. Parasites Vectors.

[15] Chaisi ME, Osinubi ST, Dalton DL, Suleman E (2019). Occurrence and diversity of avian haemosporidia in Afrotropical landbirds. Int J Parasitol Parasites Wildl.

[16] Hellgren O, Waldenström J, Bensch S (2004). A new PCR assay for simultaneous studies of Leucocytozoon, Plasmodium, and Haemoproteus from avian blood. J Parasitol.

[17] Martinsen ES, Perkins SL, Schall JJ (2008). A three-genome phylogeny of malaria parasites (Plasmodium and closely related genera): Evolution of life-history traits and host switches. Mol Phylogenet Evol.

[18] Waldenström J, Bensch S, Hasselquist D, Östman Ö (2004). A new nested polymerase chain reaction method very efficient in detecting plasmodium and haemoproteus infections from avian blood. J Parasitol.

[19] Barbosa AD, Gofton AW, Paparini A, Codello A, Greay T, Gillett A (2017). Increased genetic diversity and prevalence of co-infection with Trypanosoma spp. in koalas (Phascolarctos cinereus) and their ticks identified using next-generation sequencing (NGS). PLoS One.

[20] Bernotiene R, Palinauskas V, Iezhova T, Murauskaite D, Valkiunas G (2016). Avian haemosporidian parasites (Haemosporida): a comparative analysis of different polymerase chain reaction assays in detection of mixed infections. Exp Parasitol.

[21] Ciloglu A, Ellis VA, Bernotienė R, Valkiūnas G, Bensch S (2019). A new one-step multiplex PCR assay for simultaneous detection and identification of avian haemosporidian parasites. Parasitol Res.

[22] Pacheco MA, Cepeda AS, Bernotienė R, Lotta IA, Matta NE, Valkiūnas G (2018). Primers targeting mitochondrial genes of avian haemosporidians: PCR detection and differential DNA amplification of parasites belonging to different genera. Int J Parasitol.

[23] Paperna I, Soh M, Keong C, Yap C, May A. Haemosporozoan parasites found in birds in Peninsular Malaysia, Singapore, Sarawak and Java. RAFFLES Bull Zool. 2008;.

[24] Bensch  S, Hellgren  O, PÉrez-Tris J (2009). MalAvi: a public database of malaria parasites and related haemosporidians in avian hosts based on mitochondrial cytochrome b lineages. Mol Ecol Resour.

[25] Zehtindjiev P, Ivanova K, Mariaux J, Georgiev BB (2013). First data on the genetic diversity of avian haemosporidians in China: cytochrome b lineages of the genera Plasmodium and Haemoproteus (Haemosporida) from Gansu Province. Parasitol Res.

[26] Scaglione FE, Pregel P, Cannizzo FT, Pérez-Rodríguez AD, Ferroglio E, Bollo E (2015). Prevalence of new and known species of haemoparasites in feral pigeons in northwest Italy. Malar J.

[27] Ferraguti  M, Martínez-De La Puente  J, García-Longoria L, Soriguer  R, Figuerola  J, , Marzal  A (2019). From Africa to Europe: evidence of transmission of a tropical Plasmodium lineage in Spanish populations of house sparrows. Parasites Vectors.

[28] Levin II, Zweirs P, Deem SL, Geest EA, Higashiguchi JM, Iezhova TA (2013). Multiple lineages of avian malaria parasites ( Plasmodium ) in the Galapagos Islands and evidence for arrival via migratory birds abstract. Conserv Biol.

[29] Shazali N, Mohd-Azlan J, Tuen AA. Bird diets in urban environments: the case of the Asian Glossy Starling, Aplonis panayensis. 2016;171–81.

[30] Voon  AMF, Nasradhi  KNA, Rahman  MA, Mohd-Azlan J (2014). Bird diversity, density and foraging activities in a University campus landscape in Sarawak. Borneo J Resour Sci Technol.

[31] Sze FHD, Ab Razak NA, Mohd-Azlan J (2018). The density of invasive urban birds in selected areas of western Sarawak. Malaysian Appl Biol.

[32] Hashim NEN, Mansor MS, Abdullah NA, Ramli R (2021). The diet of a roosting population of asian glossy starling aplonis panayensis in Jelebu, Negeri Sembilan. Malaysia Sains Malaysiana.

[33] Urabe M, Nor Hashim NE, Uni S, Iwaki T, Abdullah Halim MR, Marzuki ME (2020). Description and molecular characteristics of Morishitium polonicum malayense Urabe, Nor Hashim & Uni, n. subsp. (Trematoda: Cyclocoelidae) from the Asian glossy starling, Aplonis panayensis strigata (Passeriformes: Sturnidae) in Peninsular Malaysia. Parasitol Int.

[34] Sodhi NS, Choo JPS, Lee BPYH, Quek KC, Kara AU (1997). Ecology of a mangrove forest bird community in Singapore. Raffles Bull Zool.

[35] Myers S. Birds of Borneo : Sabah, Sarawak, Brunei and Kalimantan. 2016. 1–336 p.

[36] Madeira F, Pearce M, Tivey ARN, Basutkar P, Lee J, Edbali O (2022). Search and sequence analysis tools services from EMBL-EBI in 2022. Nucleic Acids Res.

[37] Miller MA, Pfeiffer W, Schwartz T. Creating the CIPRES Science Gateway for inference of large phylogenetic trees. 2010 Gatew Comput Environ Work GCE 2010. 2010;.

[38] Tamura K, Stecher G, Kumar S (2021). MEGA11: Molecular Evolutionary Genetics Analysis Version 11. Mol Biol Evol.

[39] Bensch S, Stjernman M, Hasselquist D, Ostman O, Hansson B, Westerdahl H (2000). Host specificity in avian blood parasites: a study of Plasmodium and Haemoproteus mitochondrial DNA amplified from birds. Proc R Soc B Biol Sci.

[40] Garcia-Longoria L, Muriel J, Magallanes S, Villa-Galarce ZH, Ricopa L, Inga-Díaz WG (2022). Diversity and host assemblage of avian haemosporidians in different terrestrial ecoregions of Peru. Curr Zool.

[41] Garcia-Longoria L, Marzal A, De Lope F, Garamszegi L (2019). Host-parasite interaction explains variation in the prevalence of avian haemosporidians at the community level. PLoS One.

[42] Ishtiaq F, Beadell JS, Baker AJ, Rahmani AR, Jhala YV, Fleischer RC (2005). Prevalence and evolutionary relationships of haematozoan parasites in native versus introduced populations of common myna Acridotheres tristis. Proc R Soc B Biol Sci.

[43] Clark NJ, Olsson-Pons S, Ishtiaq F, Clegg SM (2015). Specialist enemies, generalist weapons and the potential spread of exotic pathogens: Malaria parasites in a highly invasive bird. Int J Parasitol.

[44] Muriel J, Marzal A, Magallanes S, García-Longoria L, Suarez-Rubio M, Bates PJJ (2021). Prevalence and diversity of avian haemosporidians may vary with anthropogenic disturbance in tropical habitats in Myanmar. Divers.

[45] Jiménez-Peñuela J, Ferraguti M, Martínez-de la Puente J, Soriguer RC, Figuerola J (2021). Urbanization effects on temporal variations of avian haemosporidian infections. Environ Res.

[46] Belo NO, Pinheiro RT, Reis ES, Ricklefs RE, Braga ÉM (2011). Prevalence and lineage diversity of avian haemosporidians from three distinct cerrado habitats in Brazil. PLoS One.

[47] Purse BV, Carpenter S, Venter GJ, Bellis G, Mullens BA (2015). Bionomics of temperate and tropical Culicoides midges: Knowledge gaps and consequences for transmission of Culicoides-borne viruses. Annu Rev Entomol.

[48] Wilke ABB, Vasquez C, Carvajal A, Moreno M, Fuller DO, Cardenas G (2021). Urbanization favors the proliferation of Aedes aegypti and Culex quinquefasciatus in urban areas of Miami-Dade County. Florida. Sci Reports.

[49] Hellgren O, Bensch S, Malmqvist B (2008). Bird hosts, blood parasites and their vectors - associations uncovered by molecular analyses of blackfly blood meals. Mol Ecol.

[50] Pornpanom P, Kasorndorkbua C, Lertwatcharasarakul P, Salakij C (2021). Prevalence and genetic diversity of haemoproteus and plasmodium in raptors from Thailand: data from rehabilitation center. Int J Parasitol Parasites Wildl.

[51] Pattaradilokrat S, Tiyamanee W, Simpalipan P, Kaewthamasorn M, Saiwichai T, Li J (2015). Molecular detection of the avian malaria parasite Plasmodium gallinaceum in Thailand. Vet Parasitol.

[52] Permin A, Juhl J (2002). The development of Plasmodium gallinaceum infections in chickens following single infections with three different dose levels. Vet Parasitol.

[53] Palinauskas V, Žiegyte R, Ilgunas M, Iezhova TA, Bernotiene R, Bolshakov C (2015). Description of the first cryptic avian malaria parasite, plasmodium homocircumflexum n. sp., with experimental data on its virulence and development in avian hosts and mosquitoes. Int J Parasitol.

[54] Ilgunas M, Bukauskaite D, Palinauskas V, Iezhova T, Fragner K, Platonova E (2019). Patterns of Plasmodium homocircumflexum virulence in experimentally infected passerine birds. Malar J.

[55] Platonova E, Aželytė J, Iezhova T, Ilgūnas M, Mukhin A, Palinauskas V (2021). Experimental study of newly described avian malaria parasite Plasmodium (Novyella) collidatum n. sp., genetic lineage pFANTAIL01 obtained from South Asian migrant bird. Malar J.

[56] Klem D (2014). Landscape, legal, and biodiversity threats that windows pose to birds: a review of an important conservation issue. Land.

[57] Tan DJX, Yong DL, Low BW, Owyong A, Chia A (2017). Anthropogenic sources of non-migratory avian mortalities in Singapore. Int J Trop Vet adn Biomed Res.

[58] Pendl H, Hernández-Lara C, Kubacki J, Borel N, Albini S, Valkiūnas G (2022). Exo-erythrocytic development of Plasmodium matutinum (lineage pLINN1) in a naturally infected roadkill fieldfare Turdus pilaris. Malar J.

[59] Himmel T, Harl J, Matt J, Weissenböck H (2021). A citizen science-based survey of avian mortality focusing on haemosporidian infections in wild passerine birds. Malar J.

[60] De Abreu FVS, Gomes LR, Mello ARL, Bianco-Júnior C, De Pina-Costa A, Dos Santos E (2018). Frozen blood clots can be used for the diagnosis of distinct Plasmodium species in man and non-human primates from the Brazilian Atlantic Forest. Malar J.

[61] Ciloglu A, Yildirim A, Pekmezci D, Yetismis G, Sursal Simsek N, Simsek E, et al. A novel one-step multiplex PCR protocol to detect avian haemosporidian parasites in the subgenus Haemoproteus (Kruse, 1890) used to quantify parasite prevalence in domestic pigeons (Columba livia) in Turkey. Vet Res Commun. 2022 ;1–11. Available from: https://link.springer.com/article/10.1007/s11259-022-09962-z. [cited 2023 Jan 11]. 10.1007/s11259-022-09962-z 35739341

