# Supplementary material for: Prevalence of haemosporidia in Asian Glossy Starling with discovery of misbinding of Haemoproteus-specific primer to Plasmodium genera in Sarawak, Malaysian Borneo
Source: BMC Vet Res. 2023 Apr 20;19:66. doi: 10.1186/s12917-023-03619-y (PMC10116663; doi:10.1186/s12917-023-03619-y)
Supplement: Supplementary file 1 — Additional file 1: Figure S1. Flowchart containing the workflow used for the detection of avian haemosporidians in our study. [file 12917_2023_3619_MOESM1_ESM.docx]

**Additional file 1: Figure S1.** Flowchart containing the workflow used for the detection of avian haemosporidians in our study.
