# Supplementary material for: Prevalence of haemosporidia in Asian Glossy Starling with discovery of misbinding of Haemoproteus-specific primer to Plasmodium genera in Sarawak, Malaysian Borneo
Source: BMC Vet Res. 2023 Apr 20;19:66. doi: 10.1186/s12917-023-03619-y (PMC10116663; doi:10.1186/s12917-023-03619-y)
Supplement: Supplementary file 2 — Additional file 2: Table S1. Primer name, sequence and annealing temperature used for the amplification of the three main avian haemosporidians included in our study, Plasmodium, Haemoproteus and Leucocytozoon. Primer length is included in the expected amplicon size. [file 12917_2023_3619_MOESM2_ESM.docx]

**Additional file 2: Table S1.** Primer name, sequence and annealing temperature used for the amplification of the three main avian haemosporidians included in our study, Plasmodium, Haemoproteus and Leucocytozoon. Primer length is included in the expected amplicon size.

| **PCR step** | **Targeted species** | **Primer name** | **Primer sequence**  **(5’ – 3’)** | **Expected amplicon size (bp)** | **Annealing temperature (°C)** | **Reference** |
| --- | --- | --- | --- | --- | --- | --- |
| 1^st^ round nested | All three species | **AE974-EF** | TGT AAT GCC TAG AMG WAT WCC | 1773 | 53 | Pacheco et al. (2018) |
|  |  | **AE299-ER** | GTC AAW CAA ACA TGA ATA TAG AC |  |  |  |
| 2^nd^ round nested multiplex | *Haemoproteus* | **AE980** | AAA GTT TAT TGG GWA TWY TRC CWT TAG | 346 | 57 |  |
|  |  | **AE982** | AAA CGA CCA TAT  AAA ATR WAR ATA G |  |  |  |
|  | *Plasmodium* | **AE983** | TGG ATH TGT GGW GGA TAT YTW G | 580 |  |  |
|  |  | **AE985** | AAC GAC CAT ATA WAA TGW ADA TAT C |  |  |  |
| 2^nd^ round nested | *Haemoproteus* | **HaemF** | ATG GTG CTT TCG ATA TAT GCA TG | 828 | 50 |  |
|  |  | **AE982** | AAA CGA CCA TAT AAA ATR WAR ATA G |  |  |  |
| 2^nd^ round nested | *Leucocytozoon* | **HaemFL** | ATG GTG TTT TAG ATA CTT ACA TT | 523 | 50 | Hellgren et al. (2004) |
|  |  | **HaemFR2** | CAT TAT CTG GAT GAG ATA ATG GIG C |  |  |  |
