# Supplementary material for: Prevalence of haemosporidia in Asian Glossy Starling with discovery of misbinding of Haemoproteus-specific primer to Plasmodium genera in Sarawak, Malaysian Borneo
Source: BMC Vet Res. 2023 Apr 20;19:66. doi: 10.1186/s12917-023-03619-y (PMC10116663; doi:10.1186/s12917-023-03619-y)
Supplement: Supplementary file 3 — Additional file 3: Figure S2. Uncropped electrophoresis gel of nested-multiplex PCR amplification of CytB gene of avian Plasmodium and Haemoproteus using primer set AE980/AE982 and AE983/AE985 producing amplicons of 580bp only. A positive amplification of Haemoproteus is indicated by the 346 bp amplicon in the positive control. Cropped region presented in the manuscript is denoted by the red box and labelled as Fig. 2A. [file 12917_2023_3619_MOESM3_ESM.docx]

**Additional file 3: Figure S2.** Uncropped electrophoresis gel of nested-multiplex PCR amplification of CytB gene of avian Plasmodium and Haemoproteus using primer set AE980/AE982 and AE983/AE985 producing amplicons of 580bp only. A positive amplification of Haemoproteus is indicated by the 346 bp amplicon in the positive control. Cropped region presented in the manuscript is denoted by the red box and labelled as **Figure 2A**.
