# Supplementary material for: Prevalence of haemosporidia in Asian Glossy Starling with discovery of misbinding of Haemoproteus-specific primer to Plasmodium genera in Sarawak, Malaysian Borneo
Source: BMC Vet Res. 2023 Apr 20;19:66. doi: 10.1186/s12917-023-03619-y (PMC10116663; doi:10.1186/s12917-023-03619-y)
Supplement: Supplementary file 4 — Additional file 4: Figure S3. Uncropped electrophoresis gel of nested PCR of amplification of CytB gene of avian Plasmodium using Haemoproteus-specific primer set HaemF/AE982 producing amplicons of 820bp only. Cropped region presented in the manuscript is denoted by the red box and labelled as Fig. 2B. [file 12917_2023_3619_MOESM4_ESM.docx]

**Additional file 4: Figure S3.** Uncropped electrophoresis gel of nested PCR of amplification of CytB gene of avian Plasmodium using Haemoproteus-specific primer set HaemF/AE982 producing amplicons of 820bp only. Cropped region presented in the manuscript is denoted by the red box and labelled as **Figure 2B**.
