# Supplementary material for: Prevalence of haemosporidia in Asian Glossy Starling with discovery of misbinding of Haemoproteus-specific primer to Plasmodium genera in Sarawak, Malaysian Borneo
Source: BMC Vet Res. 2023 Apr 20;19:66. doi: 10.1186/s12917-023-03619-y (PMC10116663; doi:10.1186/s12917-023-03619-y)
Supplement: Supplementary file 5 — Additional file 5: Figure S4. Uncropped electrophoresis gel of nested PCR of amplification of CytB gene of avian Leucocytozoon using nested primer set HaemFL/HaemRL2 producing amplicon of 523 bp. Cropped regions presented in the manuscript is denoted by the red box and labelled as Fig. 2C. [file 12917_2023_3619_MOESM5_ESM.docx]

**Additional file 5: Figure S4.** Uncropped electrophoresis gel of nested PCR of amplification of CytB gene of avian Leucocytozoon using nested primer set HaemFL/HaemRL2 producing amplicon of 523 bp. Cropped regions presented in the manuscript is denoted by the red box and labelled as **Figure 2C**.
